# The Unique Substance, Lidocaine and Biological Activity of the *Dioscorea* Species for Potential Application as a Cancer Treatment, Natural Pesticide and Product

**DOI:** 10.3390/plants10081551

**Published:** 2021-07-28

**Authors:** Warin Wonok, Arunrat Chaveerach, Pornnarong Siripiyasing, Runglawan Sudmoon, Tawatchai Tanee

**Affiliations:** 1 Department of Biology, Faculty of Science, Khon Kaen University, Khon Kaen 40002, Thailand; warin1104@hotmail.com (W.W.); raccha@kku.ac.th (A.C.); 2 Faculty of Science and Technology, Rajabhat Mahasarakham University, Maha Sarakham 44000, Thailand; psiripiyasing@gmail.com; 3 Faculty of Law, Khon Kaen University, Khon Kaen 40002, Thailand; rungla@kku.ac.th; 4 Faculty of Environment and Resource Studies, Mahasarakham University, Maha Sarakham 44150, Thailand

**Keywords:** *Brassica chinensis*, *Dioscorea depauperata*, *Dioscorea glabra*, HepG2, HCT-116, human hepatocyte cancer, insect bioprotectant efficiency, lidocaine

## Abstract

The six *Dioscorea* species, *D. brevipetiolata*, *D. bulbifera*, *D. depauperata* (*Dd*), *D. glabra* (*Dg*), *D. pyrifolia* and *D. hamiltonii* were analyzed for phytochemicals, toxicity in PBMCs, and biological activity in two cancer cell lines by MTT and comet assays, and pesticide efficiency. Via GC-MS, lidocaine was found to be the predominant compound in two of the studied species. To confirm the systematics, lidocaine was also found in lower amounts in 11 species. The MTT assay showed no toxicity in all six of the studied species. The comet assay showed the key result that the ethanol extracts of *Dd* and *Dg* violently broke DNA into pieces. Biological activity of these two species’ extracts showed toxicity on HepG2 and no effects on HCT-116. The water extracts of *Dd* and *Dg*, applied to *Brassica chinensis* showed high efficiency as a bioprotectant. In summary, lidocaine seems to be the predominant identifying compound of the genus *Dioscorea* in Thailand, which is useful in systematics. At least the two species, *Dd* and *Dg,* may be used for human hepatocyte cancer treatment and as an alternative pesticide for economically important vegetables. *Dioscorea* species containing lidocaine or extracted lidocaine have promise for natural product creation.

## 1. Introduction

There are 42 *Dioscorea* species in Thailand [1]. They always contain two important substances—dioscorine and the steroidal sapogenin diosgenin, which are both toxic [2,3,4]. Some species’ tubers have been used for food, for example *D. hispida*, which is very poisonous due to its dioscorine levels which cause dizziness and spasms, but special processing methods such as slicing into thin pieces, soaking them in running water for 2–3 days, and then placed in a stream to leach toxins, have been used to make them edible. The raw tuber is used as an ingredient for animal poisons, insecticides and wound medicine [5]. There are three species, *D. bulbifera*, *D. hispida*, and *D. membranacea* Pierre in Thailand recorded as traditional medicine, and one of these, *D. membranacea,* had its medicinal properties supported by a biological activity report [2]. The substance diosgenin, found in some species, has several bioactivities as reported by Jesus et al. [3] and Kumar et al. [6], including anticancer activity, anti-inflammatory, immunological activity, anti-infectious activity, effects in diabetes, dyslipidemias, and obesity, anticoagulant and antithrombotic effects, protection of cardiac cells from hypoxia–reoxygenation injury, and antioxidative effects. Aside from the tuber, bulbils or aerial bulbs have also long been used in several ways, such as *D. bulbifera* bulbils which are used in the treatment as of dysentery, syphilis, ulcers, cough, leprosy, diabetes, asthma, and cancer [2,7]. Recently, Padhan and Panda [8] revealed that *Dioscorea* species provides food and medicines in relation to their nutritional, anti-nutritional and pharmacological properties and highlights the potentiality for food and nutritional security for combating the “hidden hunger” caused by micronutrient deficiencies. Although there are many *Dioscorea* species worldwide, there is very little scientific information on them. Therefore, this research aimed to gain knowledge of certain *Dioscorea* species including phytochemicals, toxicity, biological activity, and usages in human life.

## 2. Results

### 2.1. Phytochemicals Constituent

#### 2.1.1. Gas Chromatography-Mass Spectrometry (GC-MS)

**Figure 1:**
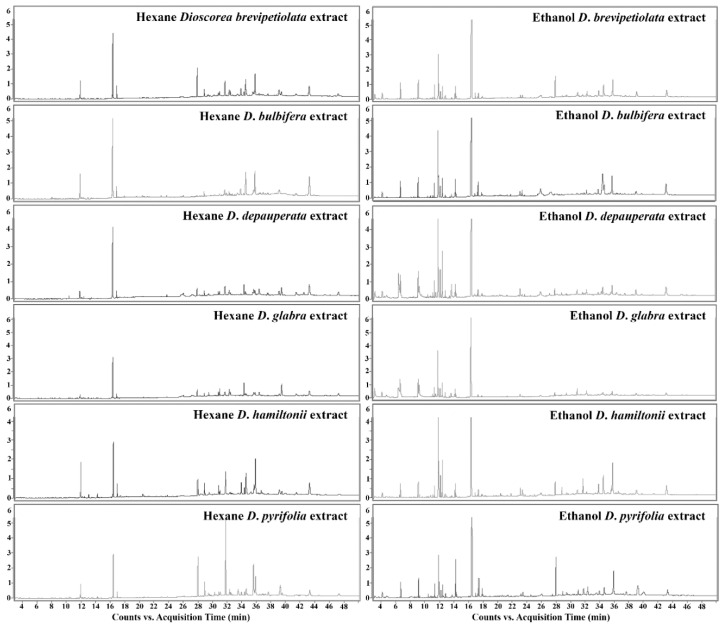
Gas chromatography-mass spectrometry chromatograms of the hexane and ethanol leaf extracts of six *Dioscorea* species showing retention time and peak areas.

Phytochemicals found in the six studied *Dioscorea* species, *D. brevipetiolata*, *D. bulbifera*, *D. depauperata*, *D. glabra*, *D. pyrifolia* and *D. hamiltonii*, by GC-MS analysis are shown in various types and quantities in Table 1 and chromatograms showing retention time and peak areas are show in Figure 1. Major quantities of phytol, γ-sitosterol, stigmasterol, and squalene were found, and minor quantities were found of other substances. The surprising finding was that the dominant substance was lidocaine, at 0.81% and 1.03% in *D. depauperata* and *D. glabra*. 

#### 2.1.2. Gas Chromatography (GC) with the Lidocaine Standard

**Figure 2:**
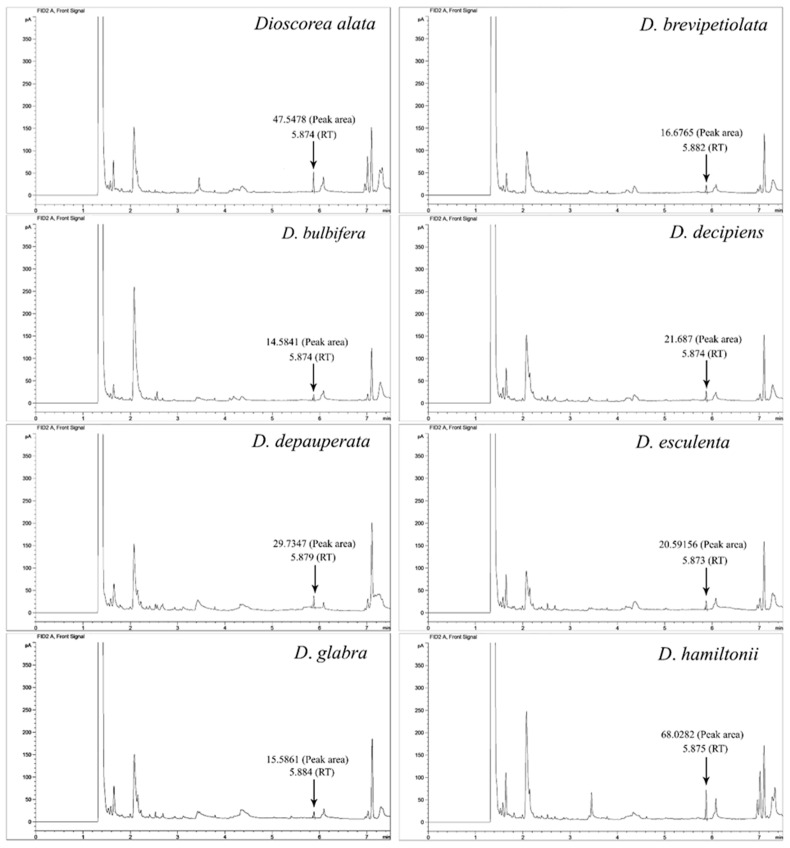
The representative peak area chromatograms of lidocaine from the eight of the 11 *Dioscorea* species studied, *D. alata*, *D. brevipetiolata*, *D. bulbifera*, *D. decipiens*, *D. depauperata*, *D. esculenta*, *D. glabra* and *D. hamiltonii*.

**Figure 3:**
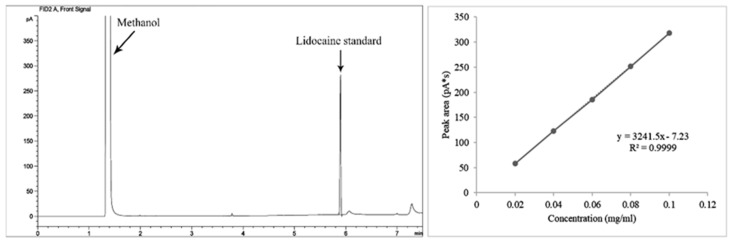
Chromatogram of the lidocaine standard and methanol solvent, and the graph of the peak area and the standard at 0.02, 0.04, 0.06, 0.08 and 0.10 mg/mL concentrations gave a correlation coefficient (R^2^) at 0.99 and linear equation, y = 3241.5x − 7.23, which was used for lidocaine content calculation of the plant extract.

When lidocaine was measured exactly by GC compared to the lidocaine standard in the 11 *Dioscorea* species, methanol extracts of *D. alata*, *D. arachidna*, *D. brevipetiolata*, *D. bulbifera*, *D. decipiens*, *D. depauperata*, *D. esculenta*, *D. glabra*, *D. hamiltonii*, *D. hispida* and *D. pentaphylla*, lidocaine content was found to range from 3.83 × 10^−3^ to 2.32 × 10^−3^ mg/mL of concentrations and 1.05 × 10^−2^ to 8.13 × 10^−2^ mg/g of plant material between *D. arachidna* and *D. hamiltonii* (Table 2), following a peak area number in the extract chromatograms (Figure 2). The chromatogram of the methanol (solvent) and lidocaine standard, plotting the peak areas and the standard concentration to create a linear equation, y = 3241.5x − 7.23 and the correlation coefficient (R^2^) at 0.99, is shown in Figure 3.

### 2.2. Toxicity

#### 2.2.1. Cytotoxicity

**Figure 4:**
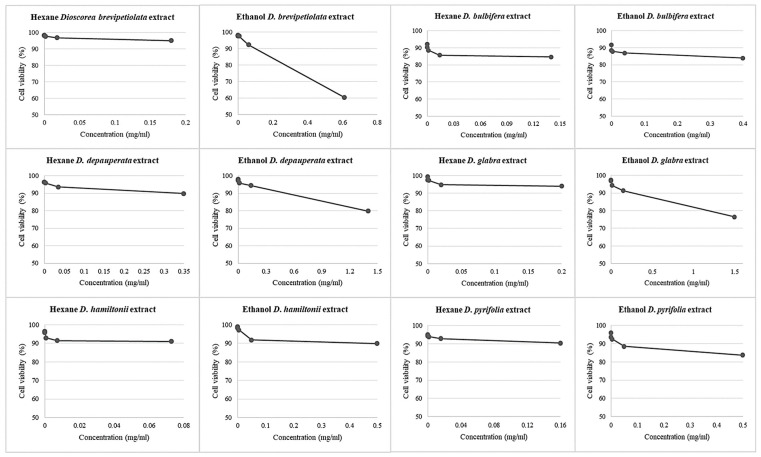
The plotting graph derived from the MTT assay, PBMC_s_ treated with two extracts of six *Dioscorea* species: hexane and ethanol extracts at various concentrations.

The maximum concentration of the hexane and ethanol extracts of the six *Dioscorea* species leaves were 10-fold diluted five times to make the working concentrations for the MTT assays on PBMCs. The results showed no toxicity on cellular levels, i.e., no IC_50_ values, with high cell viability percentages from 60.48 ± 0.07% (*D*. *bulbifera*) to 99.49 ± 0.14% (*D*. *glabra*). All details of the MTT results are shown in Figure 4 and Table 3. 

#### 2.2.2. Genotoxicity

**Figure 5:**
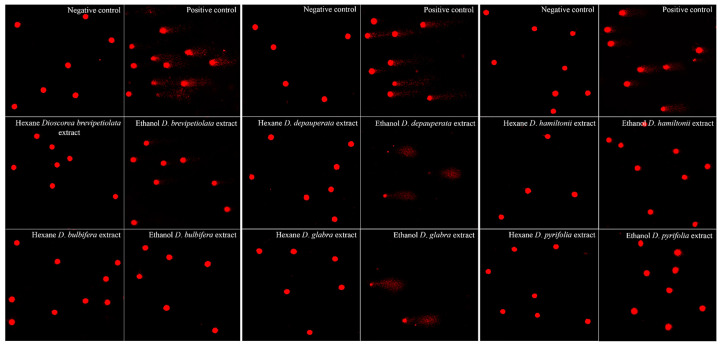
The comet assay images (200×) of PBMC_s_ treated with the highest working concentrations of the hexane and ethanol of six *Dioscorea* species extracts compared to the negative control, found from the images to have no DNA damage (all six species of hexane extracts), significant DNA damage compared to the negative control (*D. brevipetiolata*, *D. hamiltonii* and *D. pyrifolia*), DNA damage in pieces (*D. depauperata* and *D. glabra* extracts), and no DNA damage (*D. bulbifera*).

In-depth toxicity testing by comet assay with the highest working concentration which lacked IC_50_ values indicated that the six hexane extracts did not induce DNA damage. Ethanol extracts of three species, *D. brevipetiolata*, *D. hamiltonii* and *D. pyrifolia,* induced significant (*p* < 0.01) DNA damage compared to the negative control (untreated cells), and ethanol extracts of the last two species, *D. depauperata* and *D. glabra,* violently broke DNA in pieces such that the tail length cannot be measured, noting that these two ethanol *D. depauperata* and *D. glabra* extracts had higher concentrations than the other four study species, with the similar weight at 20 g in 100 mL solvent. The ethanol extract of the last species, *D. bulbifera*, did not induce DNA damage (Figure 5 and Table 4).

### 2.3. Biological Activity

**Figure 6:**
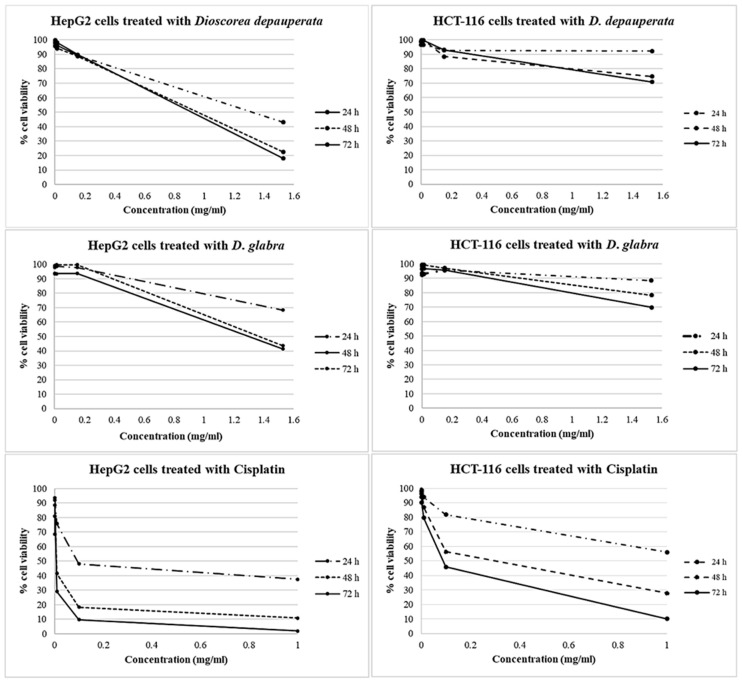
The cytotoxicity graphs showing cell viability percentages of HepG2 and HCT-116 cell lines treated with *Dioscorea depauperata*, *D. glabra* extracts and cisplatin at various concentrations and timings.

Following this, these two concentrations, the ethanol *D. depauperata* and *D. glabra* extracts, were selected for further biological activity testing on the two cancer cell lines, HepG2 and HCT-116 compared to the cisplatin control, and insecticidal efficiency. The results showed the ethanol extract of *D. depauperata* and *D. glabra* toxicity on HepG2 with IC_50_ values at 1.32 mg/mL/24 h and 1.30 mg/mL/48 h, no effects on HCT-116, and cisplatin toxicity on both HepG2 and HCT-116 at an IC_50_ value of 0.095 mg/mL/24 h and 0.29 mg/mL/48 h (Figure 6 and Table 5).

**Figure 7:**
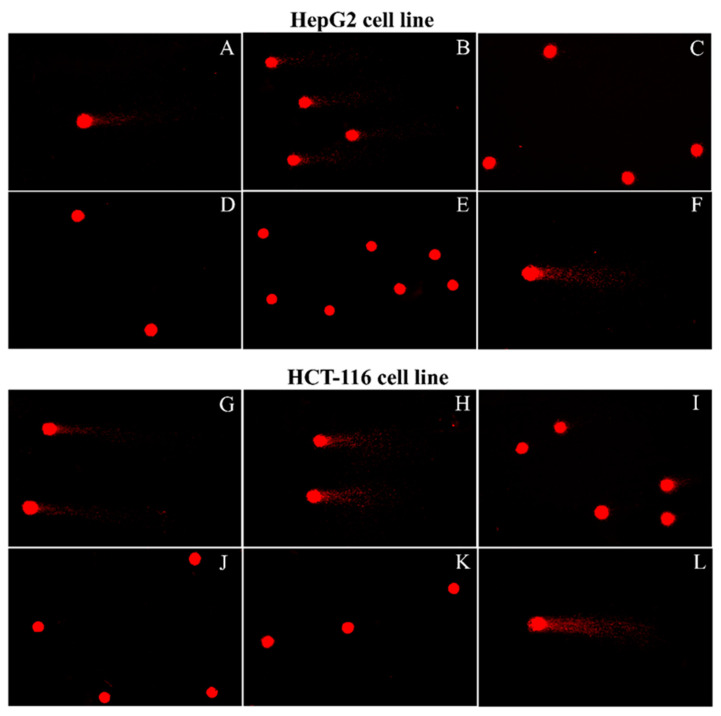
The comet assay images (200×) of HepG2 and HCT-116 cell lines treated with ethanol *Dioscorea depauperata*, *D*. *glabra* extracts and cisplatin: HepG2 cells (**A**) treated with *D*. *depauperata* for 24 h, (**B**) treated with *D*. *glabra* for 48 h, (**C**) treated with cisplatin for 24 h, (**D**) negative control for 24 h, (**E**) negative control for 48 h, (**F**) positive control; HCT-116 cells (**G**) treated with *D*. *depauperata* for 72 h, (**H**) treated with *D*. *glabra* for 72 h, (**I**) treated with cisplatin for 48 h, (**J**) negative control for 48 h, (**K**) negative control for 72 h, and (**L**) positive control.

Further, in the comet assay, these IC_50_ values of the two ethanol extracts applied to HepG2 and HCT-116 cell lines, significantly presented DNA damaged (*p* < 0.01) compared to the negative controls (the two untreated cell lines) (Figure 7 and Table 6).

### 2.4. Pesticidal Efficiency

**Figure 8:**
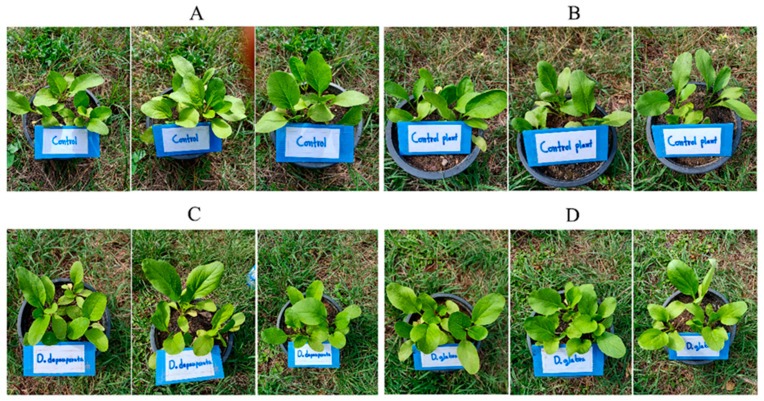
Comparison of 25-day-old *Brassica chinenesis* which were moved from the nursery to the field and sprayed with the control A (extract-untreated *Brassica chinensis*) in (**A**), control B (*Dioscorea bulbifera* extract) in (**B**), the sample C (*D. depauperata* extract) in (**C**), and sample D (*D. glabra* extract) in (**D**).

**Figure 9:**
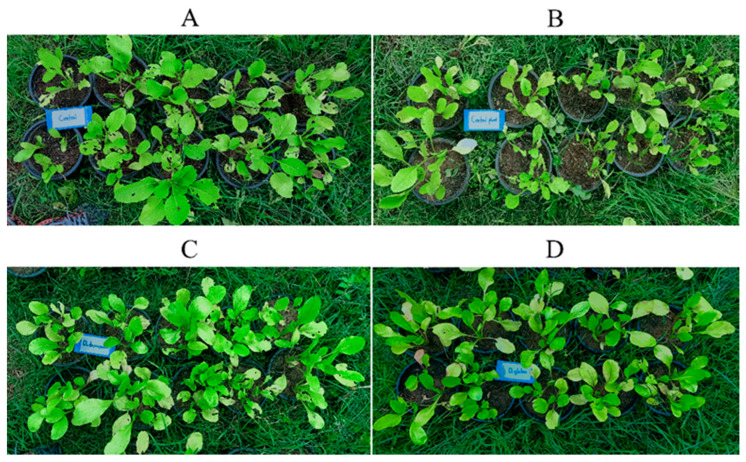
Comparison of 60-day-old *Brassica chinenesis* after the completed experiment showing the destroyed individual scoring; *B. chinenesis* was sprayed with the control A (extract-untreated *Brassica chinensis*) in (**A**), control B (*Dioscorea bulbifera* extract) in (**B**), the sample C (*D. depauperata* extract) in (**C**), and sample D (*D. glabra* extract) in (**D**).

**Figure 10:**
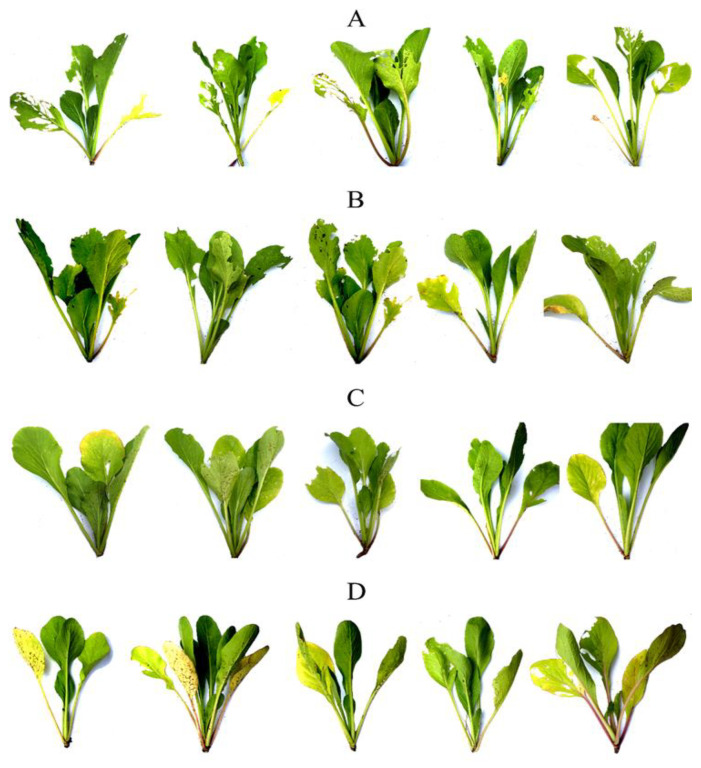
The characteristics of destroyed *Brassica chinensis* after 60 days of treatment comparing between the control extract-untreated plants (**A**), the plants treated with the extracts from *Dioscorea bulbifera* (**B**), *D. depauperata* (**C**), and *D. glabra* (**D)**.

When the 25 day-old *B. chinensis* pots (Figure 8) were transferred to the field and finished the experiment at 60 days old (Figure 9), the *B. chinensis* individuals which were destroyed by pests in an experiment (the controls A, B, and the experimental samples C and D) were counted and scored with the following results: all of the *B. chinensis* individuals of the control A, 26 of the control B, 10 of the sample C and 4 of the sample D were destroyed, scored as 30, 26, 10 and 4. All details on both destroyed individuals and characters of *B. chinensis* are shown in Figure 10 and Table 7.

## 3. Discussion

This is interesting research with new findings including lidocaine content, a unique substance expected to be part of the genus *Dioscorea’s* characteristics, and that *D. depauaperata* and *D. glabra* species can be natural pesticides and lead to anticancer drug development. Lidocaine was firstly found by GC-MS analysis in *D. depauperata* and *D. glabra* of the six studied species. The substance is very important worldwide, being used as an anesthetic in medical treatment in small amounts; quoted as an origin of modern local anesthetics [9]; broadly used in various therapeutic approaches for different types of pain, such as visceral/central pain, renal colic, and in the emergency department, since it has antinociceptive properties, turning it into a medication that is safe to administer via different routes, making it available for use in a variety of medical conditions [10]. The 10% (0.1 g/mL) lidocaine sprayed at both the oropharyngolarynx and tracheal tube cuff has a superior effect in attenuation of hemodynamic response to laryngoscopy and intubation [11]. Patients with myofascial pain in the neck and upper back are treated with a 1% (0.01 g/mL) lidocaine trigger point injection [12]. So, the discovery of lidocaine in plants should be an alternative or be used as a substitute that is both naturally sourced and is more economical than synthetics. Given this, more *Dioscorea* species, included 11 species, *D. alata*, *D. arachidna*, *D. brevipetiolata*, *D. bulbifera*, *D. decipiens*, *D. depauperata*, *D. esculenta*, *D. glabra*, *D. hamiltonii*, *D. hispida* and *D. pentaphylla* were collected for the lidocaine measurement by GC compared to the lidocaine standard, and the substance was shown in all the 11 studied species. From the larger number of species that have been studied, it can be concluded that lidocaine is a unique substance in the genus *Dioscorea*, benefitting plant systematics. Additionally, each of the species may be useful for natural product creation following previously mentioned properties—for example, ointments to relieve pain. If there was a prototype and clinical trial, it would be of great benefit to mankind. One more piece of interesting information derived from the research is that, from the two studied species, *D. depuaperata* and *D. glabra* have a selective property of being toxic to the HepG2 cancer cell line, but no toxicity to human cells compared to cisplatin activity which is an anti-cancer chemical, even though both *Dioscorea* had less anti-cancer activity than cisplatin. This result agrees with previous data reporting on substances derived from some *Dioscorea* species which have anticancer activity [3,6]. These two species with anti-cancer compounds should be experimented on in depth with a clinical trial for the further advances in cancer treatment. Additionally, the two species may be applied as an alternative pesticide for the field or garden, without hazardous effects on humans, as they have high efficiency as an insect repellent. The application method is easier than that with other plants, such as neem, which have to be fermented, whereas these two plants are simply ground, mixed with water, and then used.

Traditionally, several *Dioscorea* species have been used for the various activities mentioned in the introduction, but from the tuber or bulbil. Here, the research experimented on their leaves, which is a sustainable use of natural resources, because the leaves can always regrow.

## 4. Materials and Methods

### 4.1. Chemicals and Cell Lines

Absolute ethanol and n-Hexane AR grade were purchased from ANaPURE (New Zealand). Methanol HPLC grade, ethanol HPLC grade and dimethyl sulfoxide (DMSO) AR grade were purchased from Fisher (Loughborough, Leicestershire, UK). Lidocaine standard and 3-(4,5-dimethylthiazol-2-yl)-2,5-diphenyltetrazolium bromide (MTT) were purchased from Sigma-Aldrich (Burlington, MA, USA). RPMI 1640, with L-glutamine, Dulbecco’s Modified Eagle medium low glucose (DMEM), penicillin and trypsin were purchased from Capricorn Scientific GmbH (Ebsdorfergrund, Hesse, Germany). Ficoll-Paque Plus was purchased from GE Healthcare (Marlborough, MA, USA). Fetal bovine serum was purchased from HyClone (Marlborough, MA, USA). Hepatocellular carcinoma cell line (HepG2) and colorectal carcinoma cell line (HCT-116) were purchased from American type culture collection (ATCC, Manassas, VA, USA). Cisplatin was purchased from Fresenius Kabi (Lake Zurich, IL, USA).

### 4.2. Plant Materials and Extract Preparation

The mature leaves of the six *Dioscorea* species included *D. brevipetiolata* Prain and Burkill, *D. bulbifera* L., *D. depauperata* Prain and Burkill, *D. glabra* Roxb., *D. pyrifolia* Kunth and *D. hamiltonii* Hook.f. were collected in wild areas in Udon Thani province, northeastern Thailand (and *D. alata* L., *D. arachidna* Prain and Burkill, *D. decipiens* Hook.f., *D. esculenta* (Lour.) Burkill, *D. hispida* Dennst. and *D. pentaphylla* L. were also collected later for lidocaine detection only). They were identified following the Flora of Thailand, 2009, Dioscoreaceae. The leaves were rinsed, air-dried at room temperature for 2–3 days, then they were ground into a powder. The powder was combined with hexane or ethanol, separately at a rate 1:5, and soaked for 72 h. Each solution was filtered through a Whatman no. 1 filter paper. The filtrates were kept at −20 °C until being used in experiments including phytochemical component analysis by gas chromatography-mass spectrometry (GC-MS), 3-(4,5-dimethylthiazol-2-yl)-2,5-diphenyltetrazolium bromide (MTT), comet assays, and anticancer testing on hepatocellular carcinoma (HepG2) and colorectal carcinoma (HCT-116) cell lines.

### 4.3. Gas Chromatography-Mass Spectrometry (GC-MS)

The analysis was performed using an Agilent Technologies GC 6890 N/5973 inert mass spectrometer fused with a capillary column (30.0 m × 250 μm × 0.25 μm). Helium gas was used as the carrier at a constant flow rate of 1 mL/min. The injection and mass-transferred line temperature was set at 280 °C. The oven temperature was programmed for 70 °C to 120 °C at 3 °C/min, held isothermally for 2 min, and then raised to 270 °C at 5 °C/min. A 1 μL aliquot of the extract was injected in split mode. The relative percentage of the extract constituents was expressed as a percentage using peak area normalization. Component identification was determined by comparing the obtained mass spectra with the reference compounds in the Wiley 7N.1 library. 2.4.

### 4.4. Lidocaine Detection by Gas-Chromatography Compared to the Lidocaine Standard

Actually, lidocaine was measured in the six studied species. To be more reliable in systematics, lidocaine was measured in the extended number as 11 species. The 2 g sample leaves of the 11 studied species, *D. alata*, *D. arachidna*, *D. brevipetiolata*, *D. bulbifera*, *D. decipiens*, *D. depauperata*, *D. esculenta*, *D. glabra*, *D. hamiltonii*, *D. hispida* and *D. pentaphylla* was extracted with 10 mL methanol solvent, kept at room temperature, avoiding sunlight for 72 h. The mixtures were filtered through Whatman no.1 filter paper (125 mm diameter), then each extract was used for lidocaine detection. The chromatographic conditions were: the GC used was performed with an Agilent Technologies GC7890B, equipped with flame ionization detector (FID) and HP-5 capillary column (30.0 m × 320 μm i.d. × 0.25 μm film thickness). Helium was used as a carrier gas with a flow rate of 1.6 mL/min. The injector and detector temperatures were 260 °C. The oven temperature was programed at an initial temperature of 120 °C, held for 2 min, ramp rate of 20 °C/min and final temperature at 230 °C. The 1 μL of each sample was injected to a column at split ratio 10:1. 

Preparation of the lidocaine standard: the working solution of the standard at 20, 40, 60, 80 and 100 μg/mL was prepared in methanol. The lidocaine standard at various concentrations was injected for plotting the calibration curve. The linear equation and correlation coefficient were calculated by Microsoft Excel.

### 4.5. Cytotoxicity and Genotoxicity Testing via MTT and Comet Assays

The steps are as follows:

1. Stock Extract Preparation
  The solvents of the filtrates (from plant extract preparations) were removed with a rotary evaporator (Rotavapor R-210, Buchi, Switzerland) at 800–1000 mbar, 15 °C, and 600 rpm for 2 h. Then, dimethyl sulfoxide (DMSO) was added to the extracts until being completely dissolved and maintained as stock extracts at −20 °C conducting for the cytotoxicity and genotoxicity experiments.
2. Human Peripheral Blood Mononuclear Cells (PBMCs) Preparation
  PBMCs were isolated from sodium heparin anticoagulated venous blood from a blood bank using Ficoll-Paque Plus (GE Healthcare). Freshly isolated PBMCs with viability of at least 98% were used for the toxicity testing. The cells were suspended at a concentration of 1 × 10^6^ cells/mL for MTT and 0.4–0.6 × 10^6^ cells/mL for the comet assay in modified RPMI-1640 medium supplemented with 10% FBS, 1% antibiotic (streptomycin and penicillin).
3. MTT Assay
  The stock extract concentrations were serially 10-fold diluted with water, five times for the working concentrations. The prepared cells were seeded in 96-well plates, 125 μL per well, and 12.5 μL of the extract working concentrations were added to the corresponding wells, incubated for 24 h for PBMCs and 24, 48 and 72 h for cancer cell lines in a humidified CO_2_ incubator at 37 °C and 5% CO_2_. Corresponding DMSO concentrations were similarly prepared as vehicle controls, untreated cells and hydrogen peroxide-treated cells were the negative and positive controls, respectively. When the time was over, the plates were centrifuged at 1500 rpm for 10 min and the medium was removed, the MTT (Sigma, USA) was added to a final concentration of 0.5 mg/mL, the plates were wrapped with aluminum foil and incubated for 4 h at 37 °C. The formazan crystals were solubilized by adding 100 μL DMSO to each well, and the plates were left in the dark for 2–4 h. The absorbance was read at 570 nm with a microtiter plate spectrophotometer (Multifunction microplate reader; Varioskan Flash, Thermo fisher, Waltham, MA, USA). Wells containing medium and MTT without cells were used as blanks. Each concentration treatment was performed in triplicate. All values were expressed as the mean ± SD. Cellular reduction of MTT formed a violet crystal formazan through mitochondrial succinate dehydrogenase activity of the viable cells, and the violet crystal formazan was quantified following the methods of Freshney [13]. Percentage of cell viability was calculated using the equation cell viability (%) = average viable treated cells/average viable negative control cells × 100), to reveal the cytotoxicity of the plant extracts. Doses inducing 50% inhibition of cell viability (IC_50_ value) were determined by plotting a graph of the extract concentration against the cell viability. The IC_50_ value was used for the LD_50_ calculation Walum [14] to infer hazardous levels, according to the World Health Organization [15].
4. Comet Assay
  The concentration at IC_50_ value or the maximum-treated concentration, in the case of no IC_50_ value, was used in the comet assay to assess the genotoxicity of plant extracts, according to Singh et al. [16]. Shortly, 500 µL of cells in media was added with 50 µL extracts in a 1.5 mL microtube, incubated for 24 h for PBMCs and 24, 48 and 72 h for cancer cell lines in a humidified CO_2_ incubator at 37 °C and 5% CO_2_, then the DNA was checked by electrophoresis. The electrophoresis buffer consisted of 0.3 M NaOH and 1 mM EDTA (pH = 10). The power was supplied at a constant of 3.4 v/cm with an adjustment to 300 mA, for 25 min. To quantify the level of DNA damage, the extent of DNA migration was defined using the “Olive Tail Moment” (OTM), which is the relative amount of DNA in the tail of the comet multiplied by the median migration distance. The comets were observed at 200× magnifications and images were obtained using an image analysis system (Isis) attached to a fluorescence microscope (Nikon, Tokyo, Japan), equipped with a 560 nm excitation filter, 590 nm barrier filter, and a CCD video camera PCO (Germany). At least 150 cells (50 cells for each of triplicate slides) were examined for each experiment. The CASP software (Wroclaw, Poland) was used to analyze the OTM. The negative control was untreated cells, and the positive control was UV-treated cells. All experiments were in triplicate. The triplicate cultures were scored for the experiment. All values were expressed as the median ± S.D. The nonparametric Mann–Whitney U test was used for statistical analysis of the comet assay results; statistical significance was set at *p* < 0.05.

### 4.6. Biological Activity Testing of the Plant Extracts on HepG2 and HCT-116 Compared to Cisplatin

*Dioscorea depauperata* and *D. glabra* leaf extract (stock extract) were prepared, using the same concentration of 1.53 mg/mL, which is the highest working concentration for DNA breaking (1.42, 1.53 mg/mL, results from the MTT assay). The concentration was 10-fold diluted as 1.53 × 10^−1^, 1.53 × 10^−2^, 1.53 × 10^−3^ and 1.53 × 10^−4^ mg/mL with distilled water. Cisplatin as an anticancer chemical was prepared with a normal saline solution-derived working concentration at 1.00, 1.00 × 10^−1^, 1.00 × 10^−2^, 1.00 × 10^−3^ and 1.00 × 10^−4^ mg/mL. The substance was purchased from Srinagarind hospital, Khon Kaen University, Thailand. 

Preparations of cancer cell lines, HepG2 and HCT-116 cell lines were purchased from American type culture collection (ATCC). The cell lines were cultured by Dulbecco’s Modified Eagle medium low glucose supported with 10% fetal bovine serum and 1% antibiotic (streptomycin and penicillin). When the cells grew and flowed on the surface of the 25 cm^2^ flask, the cells were cultured and subcultured until 10 passages. The trypsin was used for trypsinization. A total of 1 mL of trypsin was added to the flask and soaked for 2–3 min. An auto pipette was used to gently suck cells and transfer them into a 15 mL tube that contained 3 mL medium and were centrifuged at 1800 rpm 5 min. The supernatant was discarded then we added 3 mL of the fresh medium into the tube. The cells were gently resuspended and 100 μL was sucked into a 1.5 mL tube. The cells were mixed with 0.1 μL erythrosine, then counted by hemocytometer. The 0.4 × 10^6^ cells/mL were used for the MTT and comet assay. After cell preparations, 125 μL of cells suspended was seeded in 96-well plates. HCT-116 was incubated at 37 °C and 5% CO_2_ for 24 h and HepG2 for 48 h. After 24 and 48 h, the culture medium was replaced with the fresh medium. Biological activity testing of the plant extracts on HepG2 and HCT-116 compared to cisplatin was performed using the MTT and comet assays. 

### 4.7. Biological Activity Testing for Pesticidal Efficiency

Pesticidal efficiency of the *Dioscorea* species extracts were tested on *Brassica chinensis*. There were four experiments with two controls. Control A is *B. chinensis* extract-untreated individuals. Control B was the plants treated with extract of *D. bulbifera*, the species which was not toxic to PBMCs on both cell and DNA levels. The sample experiments were the plants treated with extracts of *D. depauperata* (sample C) and *D. glabra* (sample D) which shown effect on DNA damage. The *B. chinensis* seeds were cultivated in 40 pots, retained in the nursery. When geminated, each pot was thinned to have three individuals. The pots were separated into four experiments of 10 pots, each experiment being the control A, control treatment B, and experimental treatment samples C and D, respectively. A total of 200 g of fresh leaves of *D. depauperata* and *D. glabra* was ground with 1 L of water solvent (at a rate 1:5) and added with the 5 mL of surfactant (tween 20). The extracts were used in the next steps or stored in a refrigerator until further used. The four groups of 10 pots each of 25-day-old *B. chinensis* were moved from the nursery outside to the field, then the three *B. chinensis* individuals of each pot were sprayed with the extracts of the controls A and B, and treatment samples C and D. The spraying was done five times, once a week. Once the *B. chinensis* reached 60 days old, they were examined for holes from insect bites, and scored as 0, 1, 2 and 3 indicating that 0, 1, 2, or 3 *B. chinensis* individuals were destroyed.

## 5. Conclusions

Lidocaine is the predominant substance of the genus *Dioscorea* in Thailand, as used in plant systematics. The two species, *D. depauperata* and *D. glabra* may be used for human hepatocyte cancer treatment, with insect protection applied as an alternative pesticide without fermenting to the vegetable. The *Dioscorea* species containing lidocaine or with extracted lidocaine can be applied to natural product creation used for medical and public health.

## Figures and Tables

**Table 1:** A summary of chemical constituents indicated by relative content percentages analyzed by gas chromatography-mass spectrometry in the six hexane (H) and ethanol (E) *Dioscorea* species leaf extracts.

Compound	Formula	Relative Content (%)											
		*D. brevipetiolata*		*D. bulbifera*		*D. depauperata*		*D*. *glabra*		*D*. *hamiltonii*		*D*. *pyrifolia*	
		H	E	H	E	H	E	H	E	H	E	H	E
Phytol	C_20_H_40_O	24.15	56.90	47.47	48.23	46.06	50.22	31.81	47.56	16.23	41.95	10.78	44.35
Phytol, acetate	C_22_H_42_O_2_	0.97	-	-	-	4.62	-	1.31	10.48	-	-	0.63	-
γ-Sitosterol	C_28_H_50_O	15.76	5.83	15.08	5.25	-	2.52	4.26	1.68	21.36	9.23	9.03	7.54
Stigmasterol	C_29_H_48_O	9.97	3.69	12.72	10.44	2.96	1.53	2.40	0.81	11.06	4.81	2.71	2.18
Squalene	C_30_H_50_	10.80	3.97	-	-	4.51	0.87	4.87	0.86	4.89	2.04	10.20	6.39
Phenol, 2-propyl-	C_11_H_17_NO_3_	-	-	-	-	-	8.35	-	10.17	-	-	-	-
Vitamin E	C_29_H_50_O_2_	6.09	2.31	4.21	1.51	5.76	-	3.79	1.45	4.69	2.23	-	2.06
Triacontanoic acid, methyl ester	C_31_H_62_O_2_	-	-	-	-	7.82	-	-	-	-	-	-	-
dl-α-Tocopherol	C_29_H_50_O_2_	3.07	1.17	1.75	0.85	3.81	0.95	6.61	1.39	-	-	2.04	1.68
2-Pentadecanone, 6,10,14-trimethyl-	C_18_H_36_O	4.27	2.15	5.46	2.75	3.16	1.32	2.00	1.10	6.75	4.19	2.18	1.91
Hexadecanoic acid, ethyl ester	C_18_H_36_O_2_	-	2.19	-	2.71	-	1.73	-	2.07	1.23	1.99	0.43	6.14
γ-Tocopherol	C_28_H_48_O_2_	1.60	0.81	-	-	2.39	0.72	5.84	1.65	1.27	0.73	0.99	0.91
Campesterol	C_28_H_48_O	4.46	1.82	3.82	1.34	-	-	-	-	6.18	2.67	1.39	1.22
1,3-Benzenediol, 4-propyl-	C_9_H_12_O_2_	-	-	-	-	-	-	-	4.90	-	-	-	-
δ-Tocopherol	C_27_H_46_O_2_	-	-	-	-	-	-	2.93	0.63	-	-	-	-
n-Hexadecanoic acid	C_16_H_32_O_2_	-	-	-	0.42	-	2.60	-	1.25	-	-	-	-
Dodecane	C_12_H_26_	-	1.67	-	1.57	-	1.47	-	2.23	-	-	-	1.58
Glycerol β-palmitate	C_19_H_38_O_4_	-	0.56	-	0.71	-	0.95	-	0.61	-	1.19	-	-
Octadecanoic acid, ethyl ester	C_20_H_40_O_2_	-	-	-	0.53	-	-	-	-	-	-	-	1.35
Tetradecane	C_14_H_30_	-	4.45	-	-	-	2.07	-	2.80	-	-	-	2.13
Benzyldiethyl-(2,6-xylylcarbamoylmethyl)- ammonium benzoate	C_28_H_34_N_2_O_3_	-	0.56	-	0.93	-	-	-	-	-	0.93	-	0.62
Lidocaine	C_14_H_22_N2_O_	-	-	-	-	-	0.81	-	1.03	-	-	-	-
Diisooctyl phthalate	C_24_H_38_O_4_	-	-	-	-	1.22	-	-	-	-	-	-	-
2-Hydroxy-5-methylisophthalaldehyde	C_9_H_8_O_3_	-	-	-	-	-	-	-	0.80	-	-	-	-
Total of identified compounds	-	81.14	88.08	90.51	77.24	82.31	76.11	65.82	93.47	73.66	71.96	40.38	80.06
Unknown	-	18.86	11.92	9.49	22.76	17.69	23.89	34.18	6.53	26.34	28.04	59.62	19.94

**Table 2:** Lidocaine measurement by GC compared to lidocaine standard resulting in concentration (μg/mL, mg/mL) and amount (mg/g plant).

Plant Extract	Retention Time (min)	Peak Area (pA*s)	Lidocaine			
			Concentration		Amount	
			μg/mL	mg/mL	mg/g	mg/100 g
*Dioscorea alata*	5.87	47.55	16.90	16.90 × 10^−3^	5.91 × 10^−2^	5.92
*D*. *arachidna*	5.87	5.18	3.83	3.83 × 10^−3^	1.05 × 10^−2^	1.05
*D*. *brevipetiolata*	5.88	16.18	7.83	7.83 × 10^−3^	2.03 × 10^−2^	2.03
*D*. *bulbifera*	5.87	14.58	6.73	6.73 × 10^−3^	1.85 × 10^−2^	1.85
*D*. *decipiens*	5.87	21.69	8.92	8.92 × 10^−3^	2.68 × 10^−2^	2.68
*D*. *depauperata*	5.88	29.73	11.40	11.40 × 10^−^^3^	3.71 × 10^−2^	3.71
*D*. *esculenta*	5.87	20.59	8.58	8.58 × 10^−3^	2.79 × 10^−2^	2.79
*D*. *glabra*	5.88	15.59	7.04	7.04 × 10^−3^	2.46 × 10^−2^	2.46
*D*. *hamiltonii*	5.88	68.03	23.22	23.22 × 10^−3^	8.13 × 10^−2^	8.13
*D*. *hispida*	5.88	14.75	6.78	6.78 × 10^−3^	2.03 × 10^−2^	2.03
*D*. *pentaphylla*	5.88	11.94	5.91	5.91 × 10^−3^	1.92 × 10^−2^	1.92

**Table 3:** The result of the MTT assay showing viability percentage of PBMC_s_ treated with hexane and ethanol extracts of six *Dioscorea* species with five working concentrations showing no toxicity, without IC_50_ values and high cell variability percentages.

Plant Extract	Solvent	Maximum Concentration (mg/mL)	Working Concentration (mg/mL)	Cell Viability (%) ± S.D.
*D. brevipetiolata*	hexane	1.81	0.18–0.18 × 10^−4^	95.09 ± 0.04–98.37 ± 0.03
	ethanol	6.15	0.61–0.61 × 10^−4^	60.48 ± 0.07–98.21 ± 0.04
*D. bulbifera*	hexane	1.46	0.14–0.14 × 10^−4^	84.62 ± 0.13–92.18 ± 0.16
	ethanol	4.00	0.40–0.40 × 10^−4^	84.01 ± 0.10–91.65 ± 0.14
*D. depauperata*	hexane	3.53	0.35–0.35 × 10^−4^	89.87 ± 0.07–96.40 ± 0.10
	ethanol	14.21	1.42–1.42 × 10^−4^	79.86 ± 0.10–97.23 ± 0.08
*D. glabra*	hexane	2.00	0.20–0.20 × 10^−4^	94.03 ± 0.07–99.49 ± 0.14
	ethanol	15.30	1.53–1.53 × 10^−4^	76.52 ± 0.09–97.30 ± 0.10
*D. hamiltonii*	hexane	0.73	0.07–0.07 × 10^−4^	91.03 ± 0.06–96.45 ± 0.12
	ethanol	5.00	0.50–0.50 × 10^−4^	89.90 ± 0.09–98.93 ± 0.09
*D. pyrifolia*	hexane	1.66	0.16–0.16 × 10^−4^	90.36 ± 0.10–95.02 ± 0.12
	ethanol	5.00	0.50–0.50 × 10^−4^	83.77 ± 0.08–96.04 ± 0.13

**Table 4:** The result of the comet assay showing the median and standard deviation of olive tail moment values of PBMC_s_ after treatment with the hexane and ethanol extracts of six *Dioscorea* species compared to the negative control. They showed no DNA damage (all six species of hexane extracts), significant DNA damage compared to the negative control (*D. brevipetiolata, D. hamiltonii* and *D. pyrifolia*), DNA damage in pieces (*D. depauperata* and *D. glabra* extracts), and no DNA damage (*D. bulbifera*).

Plant	Solvent	Median ± S.D. of Negative Control	Median ± S.D. of Treated Cell	*p* Value
*D. brevipetiolata*	hexane	0.17 ± 0.02 × 10^−2^	0.49 ± 0.01 × 10^−2^	>0.05
	ethanol		131.83 ± 0.19 × 10^−2^	<0.05
*D*. *bulbifera*	hexane		0.18 ± 0.07 × 10^−2^	>0.05
	ethanol		0.55 ± 0.02 × 10^−2^	>0.05
*D*. *depauperata*	hexane	0.14 ± 0.02 × 10^−2^	0.14 ± 0.02 × 10^−2^	>0.05
	ethanol		N/A *	N/A *
*D*. *glabra*	hexane		0.14 ± 0.01 × 10^−2^	>0.05
	ethanol		N/A *	N/A *
*D*. *hamiltonii*	hexane	0.07 ± 0.02 × 10^−2^	0.07 ± 0.01 × 10^−2^	>0.05
	ethanol		69.07 ± 0.18 × 10^−2^	<0.05
*D*. *pyrifolia*	hexane		0.07 ± 0.08 × 10^−2^	>0.05
	ethanol		0.17 ± 0.02 × 10^−2^	<0.05

* Not available.

**Table 5:** The viability percentages of HepG2 and HCT-116 cell lines treated with *Dioscorea depauperata*, *D*. *glabra* extracts and cisplatin at various concentrations and timings.

Sample	Time of Treated (Hours)	Working Concentration (mg/mL)	Cell Viability (% ± S.D.)		IC_50_ Value (mg/mL)/Time (Hours)	
			HepG2 Cell Line	HCT-116 Cell Line	HepG2 Cell Line	HCT-116 Cell Line
*D. depauperata* (Ethanol extract)	24	1.53 × 10^−4^–1.53	95.91–42.92 ± 0.07–0.07	96.74–92.36 ± 0.08–0.06	1.32/24	-
	48		99.70–22.36 ± 0.06–0.06	98.97–74.60 ± 0.08–0.10		
	72		97.97–18.01 ± 0.05–0.04	98.77–70.88 ± 0.07–0.08		
*D*. *glabra* (Ethanol extract)	24	1.53 × 10^−4^–1.53	98.38–68.27 ± 0.07–0.01	92.51–88.47 ± 0.08–0.07	1.30/48	-
	48		93.93–41.35 ± 0.07–0.08	96.56–78.28 ± 0.06–0.06		
	72		98.03–43.58 ± 0.06–0.06	98.42–69.79 ± 0.05–0.09		
Cisplatin	24	1.00 × 10^−4^–1.00	96.32–36.75 ± 0.10–0.06	97.81–55.92 ± 0.07–0.04	0.09/24	0.29/48
	48		84.07–10.20 ± 0.08–0.01	98.96–27.67 ± 0.08–0.02		
	72		81.81–2.17 ± 0.07–0.01	93.89–10.05 ± 0.07–0.02		

**Table 6:** Comet assay of HepG2 and HCT-116 cell lines showing the median and standard deviation of olive tail moment values after treatment with *Dioscorea depauperata*, *D*. *glabra* extracts and cisplatin at various concentrations and timings compared to the negative control.

Sample	Treated Cell Type	Concentration of Samples (mg/mL)	Time of Treated (Hours)	OTM (Median ± S.D.)		*p* Value of Treated Cells
				Negative Control	Treated Cell	
*D. depauperata*	HepG2	1.32	24	370.00 ± 3.60 × 10^−4^	4.021 ± 1.57	<0.01
	HCT-116	1.53	72	6.53 ± 0.22 × 10^−4^	9.050 ± 2.55	<0.01
*D*. *glabra*	HepG2	1.30	48	230.00 ± 3.20 × 10^−4^	12.743 ± 2.39	<0.01
	HCT-116	1.53	72	6.53 ± 0.22 × 10^−4^	11.942 ± 3.05	<0.01
cisplatin	HepG2	0.09	24	370.00 ± 3.60 × 10^−4^	0.703 ± 0.19	<0.01
	HCT-116	0.29	48	6.28 ± 0.54 × 10^−4^	0.447 ± 0.21	<0.01

**Table 7:** The results of pesticidal efficiency including four experiments (control A, B and sample C, D), where each experiment comprised 10 pots, each pot comprised 3 individuals, the individuals were scored as 1, 2, 3 when they were destroyed/bitten by a pest, 0 as if no individual was destroyed. Control A = extract-untreated *Brassica chinensis*, *B. chinensis* treated with extracts from *Dioscorea bulbifera* (Control B), sample C = *D. depauperata* (sample C)*,* and *D. glabra* (sample D).

Experiment	Number of Destroyed *Brassica chinensis* Individuals in a Pot										
	Pot 1	Pot 2	Pot 3	Pot 4	Pot 5	Pot 6	Pot 7	Pot 8	Pot 9	Pot 10	Total
Control A	3	3	3	3	3	3	3	3	3	3	30
Control B	1	3	3	3	3	3	3	2	3	2	26
Sample C	1	1	0	1	1	2	1	1	1	1	10
Sample D	1	0	0	1	1	0	0	0	0	1	4

## Data Availability

Not applicable.

## Abbreviations

PBMCs	human peripheral blood mononuclear cells
MTT	3-(4,5-dimethylthiazol-2-yl)-2,5-diphenyltetrazolium bromide
GC-MS	gas chromatography-mass-spectrometry
HepG2	hepatocellular carcinoma cell line
HCT-116	colorectal carcinoma cell line
